# Supplementary material for: Age-dependent changes in protein incorporation into collagen-rich tissues of mice by in vivo pulsed SILAC labelling
Source: eLife. 2021 Sep 28;10:e66635. doi: 10.7554/eLife.66635 (PMC8478409; doi:10.7554/eLife.66635)
Supplement: Transparent reporting form [file elife-66635-transrepform1.docx]

***eLife’s* transparent reporting form**

We encourage authors to provide detailed information *within their submission* to facilitate the interpretation and replication of experiments. Authors can upload supporting documentation to indicate the use of appropriate reporting guidelines for health-related research (see [EQUATOR Network](http://www.equator-network.org/%20)), life science research (see the [BioSharing Information Resource](https://biosharing.org/)), or the [ARRIVE guidelines](http://www.plosbiology.org/article/info:doi/10.1371/journal.pbio.1000412) for reporting work involving animal research. Where applicable, authors should refer to any relevant reporting standards documents in this form.

If you have any questions, please consult our Journal Policies and/or contact us: [editorial@elifesciences.org](mailto:editorial@elifesciences.org).

**Sample-size estimation**

- You should state whether an appropriate sample size was computed when the study was being designed
- You should state the statistical method of sample size computation and any required assumptions
- If no explicit power analysis was used, you should describe how you decided what sample (replicate) size (number) to use

Please outline where this information can be found within the submission (e.g., sections or figure legends), or explain why this information doesn’t apply to your submission:

At the time of designing this study, there were no previous publications on *in vivo* pulsed SILAC labeling in solid tissues. We took an a priori and pragmatic decision to use n=4 mice per data point and optimized our chance of seeing label incorporation by (1) performing a pilot study in skeletally immature mice (where synthetic rates were highest) and (2) labeling for a three week period. The data we generated showed that greater than 70% of proteins identified had heavy label incorporation, there was good range of % incorporation across the different proteins and tissues (30-97%, shown in Figure2-source data 1), and high reproducibility (shown in whisker plots, Figure 2-Figure supplement 1). In view of this, we determined that this number of samples, using this labeling protocol, would give us optimal chance of seeing regulation of new protein incorporation at the later time points.

**Replicates**

- You should report how often each experiment was performed
- You should include a definition of biological versus technical replication
- The data obtained should be provided and sufficient information should be provided to indicate the number of independent biological and/or technical replicates
- If you encountered any outliers, you should describe how these were handled
- Criteria for exclusion/inclusion of data should be clearly stated
- High-throughput sequence data should be uploaded before submission, with a private link for reviewers provided (these are available from both GEO and ArrayExpress)

Please outline where this information can be found within the submission (e.g., sections or figure legends), or explain why this information doesn’t apply to your submission:

Each proteomic experiment was performed once, using 4 biological replicates, looking at 4 tissues at 3 postnatal developmental stages. This is stated in Methods and number of replicates are stated in each figure legend. Details of the statistical methodology, which includes normalization, imputation and analysis are described in the main manuscript Material and Methods section. Our authorship team includes experts in proteomics, bioinformatics and statistics.

**Statistical reporting**

- Statistical analysis methods should be described and justified
- Raw data should be presented in figures whenever informative to do so (typically when N per group is less than 10)
- For each experiment, you should identify the statistical tests used, exact values of N, definitions of center, methods of multiple test correction, and dispersion and precision measures (e.g., mean, median, SD, SEM, confidence intervals; and, for the major substantive results, a measure of effect size (e.g., Pearson's r, Cohen's d)
- Report exact p-values wherever possible alongside the summary statistics and 95% confidence intervals. These should be reported for all key questions and not only when the p-value is less than 0.05.

Please outline where this information can be found within the submission (e.g., sections or figure legends), or explain why this information doesn’t apply to your submission:

All details about statistical analysis methods are reported in “Statistical analysis” section under Material and Methods, as well as in the relevant figure legends.

(For large datasets, or papers with a very large number of statistical tests, you may upload a single table file with tests, Ns, etc., with reference to sections in the manuscript.)

**Group allocation**

- Indicate how samples were allocated into experimental groups (in the case of clinical studies, please specify allocation to treatment method); if randomization was used, please also state if restricted randomization was applied
- Indicate if masking was used during group allocation, data collection and/or data analysis

Please outline where this information can be found within the submission (e.g., sections or figure legends), or explain why this information doesn’t apply to your submission:

All mice were genetically homogeneous (C57BL6J) and purchased from Charles River laboratories, UK. Mice from experimental groups A and B were taken from a breeding colony of these mice within our animal unit. C and D were purchased directly for the experiment. No pre-selection of mice was performed and only four mice were labeled for each time point. Four animals per cage was maintained to control for mouse activity levels, which could have been a confounder. The animal operative was not blinded to the experimental group as it was not possible to mix ages of mice within cage. Samples were injected into the mass spectrometer by a blinded operative. The bioinformatician designed a data analysis algorithm that was applied in a non-bias fashion across the datasets.

**Additional data files (“source data”)**

- We encourage you to upload relevant additional data files, such as numerical data that are represented as a graph in a figure, or as a summary table
- Where provided, these should be in the most useful format, and they can be uploaded as “Source data” files linked to a main figure or table
- Include model definition files including the full list of parameters used
- Include code used for data analysis (e.g., R, MatLab)
- Avoid stating that data files are “available upon request”

Please indicate the figures or tables for which source data files have been provided:

Source data files are provided for all figures. Python libraries from the Clinical Knowledge Graph’s analytics core (Santos, A. et al) were used for visualization and statistical analysis. Proteomics raw file are deposited in PRIDE repository, with reference provided in “Protein identification” under Materials and Methods section.
